# A critical role of brain network architecture in a continuum model of autism spectrum disorders spanning from healthy individuals with genetic liability to individuals with ASD

**DOI:** 10.1038/s41380-022-01916-w

**Published:** 2022-12-27

**Authors:** Budhachandra Khundrakpam, Neha Bhutani, Uku Vainik, Jinnan Gong, Noor Al-Sharif, Alain Dagher, Tonya White, Alan C. Evans

**Affiliations:** 1 McGill Centre for Integrative Neuroscience, Montreal Neurological Institute, McGill University, Montreal, QC H3A 2B4 Canada; 2 Montreal Neurological Institute, McGill University, Montreal, QC H3A 2B4 Canada; 3 Institute of Psychology, Faculty of Social Sciences, University of Tartu, Naituse 2-216, 50409 Tartu, Estonia; 4 School of Computer Science, Chengdu University, Chengdu, China; 5 Erasmus MC-Sophia/Kamer KP-2869, Postbus 2060, 3000 CB Rotterdam, Netherlands

**Keywords:** Neuroscience, Genetics, Autism spectrum disorders

## Abstract

Studies have shown cortical alterations in individuals with autism spectrum disorders (ASD) as well as in individuals with high polygenic risk for ASD. An important addition to the study of altered cortical anatomy is the investigation of the underlying brain network architecture that may reveal brain-wide mechanisms in ASD and in polygenic risk for ASD. Such an approach has been proven useful in other psychiatric disorders by revealing that brain network architecture shapes (to an extent) the disorder-related cortical alterations. This study uses data from a clinical dataset—560 male subjects (266 individuals with ASD and 294 healthy individuals, CTL, mean age at 17.2 years) from the Autism Brain Imaging Data Exchange database, and data of 391 healthy individuals (207 males, mean age at 12.1 years) from the Pediatric Imaging, Neurocognition and Genetics database. ASD-related cortical alterations (group difference, ASD-CTL, in cortical thickness) and cortical correlates of polygenic risk for ASD were assessed, and then statistically compared with structural connectome-based network measures (such as hubs) using spin permutation tests. Next, we investigated whether polygenic risk for ASD could be predicted by network architecture by building machine-learning based prediction models, and whether the top predictors of the model were identified as disease epicenters of ASD. We observed that ASD-related cortical alterations as well as cortical correlates of polygenic risk for ASD implicated cortical hubs more strongly than non-hub regions. We also observed that age progression of ASD-related cortical alterations and cortical correlates of polygenic risk for ASD implicated cortical hubs more strongly than non-hub regions. Further investigation revealed that structural connectomes predicted polygenic risk for ASD (*r* = 0.30, *p* < 0.0001), and two brain regions (the left inferior parietal and left suparmarginal) with top predictive connections were identified as disease epicenters of ASD. Our study highlights a critical role of network architecture in a continuum model of ASD spanning from healthy individuals with genetic risk to individuals with ASD. Our study also highlights the strength of investigating polygenic risk scores in addition to multi-modal neuroimaging measures to better understand the interplay between genetic risk and brain alterations associated with ASD.

## Introduction

Neuroimaging studies have consistently shown alterations in cortical anatomy in individuals with autism spectrum disorders (ASD) [1–10]. For example, a recent large-scale study out of the ENIGMA consortium (3222 individuals, 1571 with AUTISM) showed increased cortical thickness in the frontal cortex (Cohen’s *d* = 0.20) and decreased thickness in the temporal cortex (Cohen’s *d* = −0.21) in individuals with ASD [7]. Interestingly, the ASD-related cortical alterations extend beyond those with clinical diagnosis to the general population [11–14], consistent with an emerging framework that conceptualizes ASD as a continuum model (with a normal distribution of autistic tendencies in the general population where a full diagnosis is at the severe tail of the distribution [12, 13, 15–21]). For example, cortical thickness of the frontal and parietal regions were dimensionally related to genetic risk for ASD in general population, and were also part of the cortical alterations associated with ASD in clinical population [22].

An important addition to the study of altered cortical anatomy in ASD is the investigation of the underlying brain network architecture that may reveal brain-wide mechanisms in ASD. Such an approach has been proven useful in other psychiatric disorders by revealing that brain network architecture shapes (to an extent) the disorder-related morphological alterations [23, 24]. Of particular interest is the study of brain *hubs* (regions with several connections that serve as information relay centers [25, 26]) which have been implicated in several psychiatric disorders [23, 27–29]. These studies revealed that cortical alterations associated with the disorders were greater in the hubs compared to the peripheral regions (with only local connections), possibly due to the high metabolic activity and their links with several brain networks [24, 27, 30]. In addition to examining hubs, recent studies have also identified *disease epicenters*, defined as regions whose network architecture play central role in the whole-brain manifestation of psychiatric disorders [23, 24, 31–33]. Taken together, investigation of hubs and disease epicenters may provide novel insights into how patterns of ASD-related cortical alterations may be configured by the brain network architecture.

We, therefore, set out to examine the relation between brain network architecture and ASD-related alterations in cortical anatomy. Extending this goal in the context of the continuum model, we also set out to examine the link between brain network architecture and cortical correlates of polygenic risk for ASD. For this, (i) ASD-related cortical alterations (group difference, ASD-CTL in cortical thickness) using a clinical cohort and (ii) cortical correlates of polygenic risk for ASD using a general population, were first computed. Next, using normative structural brain networks derived from diffusion magnetic resonance imaging (dMRI) data from general population, we then tested the hypothesis—whether there was a selective vulnerability of hub regions that parallel the ASD-related cortical alterations as well as the cortical correlates of polygenic risk for ASD. Lastly, we set out to investigate whether polygenic risk for ASD could be predicted by structural brain networks, and whether the top predictors of the model corresponded with the disease epicenters of ASD.

## Methods

### Subjects

Data for the study were taken from two publicly available databases: (i) a clinical cohort: the Autism Brain Imaging Data Exchange (ABIDE) database [34], and (ii) a general population cohort: the Pediatric Imaging, Neurocognition and Genetics (PING) study [35]. While ABIDE is an agglomerative dataset of MRI scans of healthy individuals and individuals with ASD [34], the PING study comprises of neuroimaging, cognition and genetic data from 1493 typically developing children and adolescents collected from ten different sites across the United States [35]. As mentioned in [35], written parental informed consent was obtained for all PING individuals below the age of 18, and for all PING individuals between the ages of 7 and 17, child assent was obtained.

### Genomic data and computation of polygenic risk scores

The PING dataset includes 550,000 single nucleotide polymorphisms (SNPs) genotyped from saliva samples using Illumina Human660W-Quad BeadChip. Computation of polygenic risk scores (PRS) followed steps similar to that of our previous study [22]. Summary of steps include: preparation of the data for imputation using the “imputePrepSanger” pipeline (https://hub.docker.com/r/eauforest/imputeprepsanger/) and implemented on CBRAIN [36] using Human660W-Quad_v1_A-b37-strand chip as reference. The next step involved data imputation with Sanger Imputation Service [37], using default settings and the Haplotype Reference Consortium, HRC (http://www.haplotype-reference-consortium.org/) as the reference panel. Using Plink 1.9 [38], the imputed SNPs were then filtered with the inclusion criteria: (i) SNPs with unique names, (ii) only ACTG and (iii) MAF > 0.05. All SNPs that were included had INFO scores *R*^*2*^ > 0.9 with Plink 2.0. Next, using polygenic score software PRSice 2.1.2 [39] additional ambiguous variants were excluded, resulting in 4,696,385 variants being available for polygenic scoring. We filtered individuals with 0.95 loadings to the European principal component (GAF_Europe variable provided with the PING data), resulting in 526 participants. These participants were then used to compute 20 principal components with Plink 1.9. The polygenic risk score for ASD was based on ASD GWAS trained on 18,381 independent individuals with ASD and 27,969 controls [40]. Similar to our previous study [22], the data were clumped based on PRSice default settings (clumping distance = 250 kb, threshold *r*^2^ = 0.1), using *p* = 0.001 cut-off criterion. After matching with available variants in the data, the polygenic risk score for ASD was based on 1245 variants.

### Image pre-processing and quality control

Details of image acquisition are included in Supplementary Material S1. For structural MRI data (of both the ABIDE and PING datasets), we used the CIVET processing pipeline, (https://mcin.ca/technology/civet/) developed at the Montreal Neurological Institute to compute cortical thickness measurements at 81,924 regions covering the entire cortex. Summary of steps include; non-uniformity correction of the *T*_*1*_-weighted image and then linear registration to the Talairach-like MNI152 template (created with the ICBM152 dataset). After repeating the non-uniformity correction using template mask, the non-linear registration from the resultant volume to the MNI152 template is computed, and the transform used to provide priors to segment the image into GM, WM, and cerebrospinal fluid. Inner and outer GM surfaces are then extracted using the Constrained Laplacian-based Automated Segmentation with Proximities algorithm. Cortical thickness is then measured in native space using the linked distance between the two surfaces at 81,924 vertices. In order to impose a normal distribution on the corticometric data and to increase the signal to noise ratio, each individual’s cortical thickness map was blurred using a 30 millimeter full width at half maximum surface-based diffusion smoothing kernel. Two independent reviewers performed quality control (QC) of the data, and only scans with consensus of the two reviewers were used. Exclusion criteria for QC procedure include - data with motion artifacts, a low signal to noise ratio, artifacts due to hyperintensities from blood vessels, surface-surface intersections, or poor placement of the gray or white matter (GM and WM) surface for any reason. Details of QC procedure are included in Supplementary Material S2.

For diffusion MRI data (of the PING dataset), we used the FSL pipeline (FMRIB Software Library v5.0.9) [41] for pre-processing. Summary of steps include; correction of the distortion effects induced by eddy currents, inter-volume movements and susceptibility of the diffusion data; rigid alignment of the individual unweighted image with the structural image using flirt; non-linear registration to transform individual structural image to an MNI152 standard T_1_-weighted template using fnirt; computing the forward and backward warp field images between individual dMRI and MNI T_1_ spaces by concatenating (or inverting) the rigid transformation matrix and the warp field image. Diffusion parameters at each voxel were estimated by using Markov Chain Monte Carlo (MCMC) sampling [42, 43]. Details of the MCMC sampling are given in Supplementary Material S3. In this step, up to 2 possible fiber populations were modeled for each voxel after 2000 iterations. Quality control of the data was performed by checking the structural image and the average of the non-diffusion-weighted images for each individual, and then evaluation of the results of registration by visual inspection. Exclusion criteria of QC include – if signal-noise-rate of structural image or unweighted-diffusion image was lower than 800, and data with >2 mm frame-wise displacements of the dMRI.

### Sample characteristics

For the clinical cohort (ABIDE dataset), apart from the QC procedure, there were additional exclusion criteria namely insufficient number of individuals in each diagnostic group (ASD and CTL) to determine group difference, too few females resulting to excluding all females, and excluding individuals over 35 years of age due to insufficient numbers. The final sample consisted of 560 male individuals, 266 individuals with ASD (17.2 ± 6.4 years) and 294 controls (17 ± 6.4 years) (Table 1A). The cortical thickness data for both ASD and CTL groups followed normal distribution (Supplementary Material S6). In terms of variation of the cortical thickness data within groups, there was no significant difference (*Fstat* = 1.02, *p* = 0.82) between the variance of ASD data (var = 0.0187) and variance of CTL data (var = 0.0182).

**Table 1:** Description of study sample.

A. ABIDE sample		B. PING sample	
Number of subjects, *N*	560	Number of subjects, *N*	391
Males	560	Males/females	207/184
ASD/CTL	266/294	Age (in years)	12.1 ± 4.7
Age-ASD (in years)	17.2 ± 6.4	PRS	5.5e-03 ± 5.8e-04
Age-CTL (in years)	17.0 ± 6.4	NTCB Reading score	134.4 ± 70.6
FSIQ-ASD	106.4 ± 15.8	NTCB Flanker score	7.8 ± 1.7
FSIQ-CTL	111.4 ± 12.1	NTCB DCCS score	7.8 ± 1.3

A) Clinical sample from the ABIDE dataset, (B) General population sample from the PING dataset. Note, age is given as mean ± std, ABIDE = Autism Brain Imaging Data Exchange, PING = Pediatric Imaging, Neurocognition and Genetics, PRS = Polygenic risk score, FSIQ = full-scale intelligence quotient, NTCB = The NIH Toolbox Cognition Battery.

For the general population (PING dataset), of the total 1493 individuals, filtering for individuals with 0.95 loadings to the European principal component resulted in 526 individuals. Of these, 95 individuals did not have MRI data and 2 subjects did not have information about age, resulting in 429 subjects. Next, 13 subjects were excluded before any processing (raw data) due to severe motion and slicing artifacts. A subsequent 25 subjects failed CIVET pipeline (for a number of reasons including presence of bright blood vessels and poor contrast) and were excluded in further analysis. The final sample consisted of 391 participants (males/females = 207/184, age = 12.1 ± 4.7 years) (Table 1B). The cortical thickness data for PING as well as the PRS-ASD data followed normal distribution (Supplementary Material S6).

### Determination of structural connectivity matrices

Structural connectivity between two brain regions was computed as the mean of the streamlines between the two regions. There were 62 regions of interest (ROI), and as such the resulting structural connectivity matrix was (62 × 62). The list of ROIs are included in Supplementary Material S5. The ventricles were used as exclusion mask. Zero connections were not omitted. The subcortical regions were removed prior to analysis. The fiber counts were not log-transformed.

### Determination of hubs

The structural connectivity matrices were used to determine hubs (regions with several connections that serve as information relay centers) by computing centrality maps based on graph-theoretic measures (e.g., degree centrality) [44]. In one of the pioneer papers on the definition of hubs in the field of neuroscience, Sporns et al. identified hubs and characterized their network contributions by examining centrality indices for all regions within the cerebral cortices [44]. Measures of centrality (including degree, betweenness, closeness, eigenvector and pagerank centrality) identified brain regions that lie on many of the shortest paths between parts of the network. Among these, the simplest graph measure that has been used for identifying hubs is the node degree, also called degree centrality, which is equal to the number of edges that are maintained by each node [26]. In terms of empirical results, degree centrality has shown a central role for the precuneus and superior frontal gyrus [45], findings shown to be consistent with classic work on the functional importance of these regions. With relevance to the current manuscript, recent studies have shown the utility of degree centrality in inferring nodal stress models for brain disorders such as epilepsy [46]. Nodal stress models were derived from spatial correlation analyses between cortical syndrome-specific atrophy profiles and normative weighted degree centrality maps. In consistent with these studies, we used degree centrality to define hubs and correspondingly in the analysis of nodal stress models. Computations were done using the Brain Connectivity Toolbox https://sites.google.com/site/bctnet/.

### Statistical analysis

#### General linear model for group difference (ASD-CTL) in cortical thickness (using ABIDE dataset)

As in our previous publication [4], we built a general linear model (GLM) for finding the group difference (ASD-CTL) in cortical thickness. Cortical thickness was modeled as:

$$T_i = intercept + \beta _1Site + \beta _2Group + \beta _3Age + \varepsilon _i$$

where *i* is a vertex, *Age* is mean-centered, *ε* is the residual error, Group denotes the diagnostic groups (autism spectrum disorder, ASD and controls, CTL), and the intercept and the *β* terms are the fixed effects. Several studies have shown significant effects of age and site/scanner on cortical thickness [8, 47–49], and therefore they are included as covariates in the GLM. All statistical analyses were done with GLMs using the SurfStat toolbox (http://www.math.mcgill.ca/keith/surfstat/).

#### General linear model for the association of polygenic risk for ASD and cortical thickness (using PING dataset)

Similar to our previous publication [22], cortical thickness was modelled as:

$$T_i = intercept + \beta _1Age + \beta _2PRS + \beta _3PC20 + \beta _4Scanner + \beta _5Sex + \varepsilon _i$$

where *i* is a vertex, *Age* is mean-centered, *ε* is the residual error, PRS is the polygenic risk score, and the intercept and the *β* terms are the fixed effects. To minimize the chance of population structure explaining the polygenic score results, we extracted 20 first principal components (PC20) and used them as covariates. Without controlling for those principal components, random differences in population genomic signature can explain outcomes, if different populations also happen to differ in the outcome [50]. Since there were 9 sites but 13 scanners, device serial number (unique for each scanner, provided in PING) was put as covariate in the analyses.

#### General linear model for interaction of age and group difference (ASD-CTL) in cortical thickness (using ABIDE dataset)

As in our previous publication [4], we built a GLM for finding the effect of age on group difference (ASD-CTL) in cortical thickness. Cortical thickness was modeled as:

$$T_i = intercept + \beta _1Site + \beta _2Group + \beta _3Age + \beta _4\left( {Age \times Group} \right) + \varepsilon _i$$

where *i* is a vertex, *Age* is mean-centered, *ε* is the residual error, Group denotes the diagnostic groups (ASD and CTL), and the intercept and the *β* terms are the fixed effects.

#### General linear model for the association of polygenic risk for ASD and cortical thickness (using PING dataset)

Similar to our previous publication [22], cortical thickness was modelled as:

$$T_i = \; 	intercept + \beta _1Age + \beta _2PRS + \beta _3PC20 + \beta _4Scanner + \beta _5Sex \\ 	+ \beta _4\left( {Age \times PRS} \right) + \varepsilon _i$$

where *i* is a vertex, *Age* is mean-centered, *ε* is the residual error, PRS is the polygenic risk score, and the intercept and the *β* terms are the fixed effects.

Instead of including the covariates in our GLM analyses, we also ran ComBat [51] on the raw cortical thickness data. The resulting data was then used to perform all the GLM analyses as before.

### Statistical analysis of regional overlap

Since hub regions have been shown to be more susceptible to disorder-related cortical alterations than non-hub regions, we next investigated whether the observed cortical differences were influenced by the network connectivity of brain regions. For this, we statistically checked regional overlap between (i) map of the group difference (ASD-CTL) in cortical thickness (using the ABIDE dataset) and (ii) centrality map. In a similar manner, we statistically checked regional overlap between (i) map of the association of polygenic risk for ASD and cortical thickness (using the PING dataset) and (ii) centrality map. The statistical comparisons were done using the spin test developed by [52]. In short, the method, using a spatial permutation framework, generates null models of overlap by applying random rotations to spherical representations of the cortical surface. As in previous studies [53], 1000 surface rotations of the PING map were generated, and the statistical overlap was checked by comparing whether the observed cross-vertex correlation between two maps was statistically greater (*p* < 0.05) than those with 1000 rotations. Similar analysis was done for statistically comparing the centrality maps and (i) map of age*(ASD-CTL) on cortical thickness (using the ABIDE dataset) and (ii) map of age*(PRS) on cortical thickness (using the PING dataset).

### Connectome predictive modeling of polygenic risk for ASD

With structural connectivity data as input, we used the connectome predictive modeling (CPM) approach to investigate whether structural connectomes can be used to predict PRS-ASD. Recently introduced, CPM approach [54, 55] is a data-driven framework which utilizes cross-validation to build predictive models of brain-behavior associations from connectivity data. Comprising of four steps—feature selection, feature summarization, model building and computation of prediction significance [54, 55], the framework has been validated and used in predicting anxiety [56], attention [57], maternal bonding [58], etc. The accuracy of the prediction model was assessed by computing correlation between the true and predicted PRS-ASD scores. Next, the top predictors of the model were visualized using the BrainNet Viewer [59].

### Computation of disease epicenters of ASD

In order to check whether the top predictors of the model were identified as disease epicenters of ASD, we first compared each brain region’s structural connectivity with the ASD-related cortical alterations. Next, we used spin permutation tests to compute the significance of the correlations. Brain regions with statistically significant correlations were identified as disease epicenters of ASD. Lastly, we checked whether any of the identified disease epicenters of ASD corresponded with the top predictors of PRS-ASD.

#### Power analysis

For the ABIDE dataset, we performed a power analysis and found that, based on an alpha level of 0.05 (two-tailed test), a sample size of 253 individuals with ASD was needed to yield an estimated power of at least 0.80 to compute group difference (ASD-CTL) in cortical thickness. For the PING dataset, based on our previous publication [22], we estimated a correlation of *r* = 0.2 between PRS-ASD and cortical thickness. For this *r* value (effect), power for the sample (*N* = 391) was 0.97. Power analysis was performed using ‘*sampsizepwr*’ and ‘*binofit*’ in MATLAB.

## Results

### Hub organization is associated with ASD-related cortical alterations as well as cortical correlates of polygenic risk for ASD

**Fig. 1:**
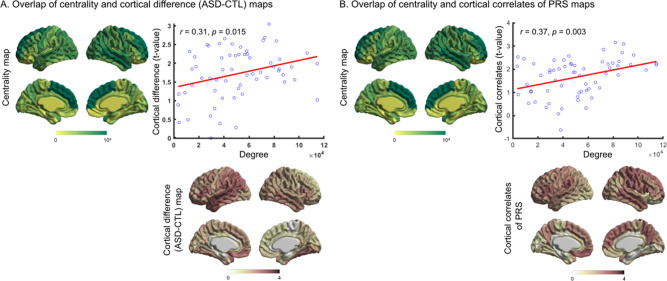
Association of structural hub organization and ASD-related cortical alterations and cortical correlates of polygenic risk for ASD. **A** Significant correlation (*r*_spin_ = 0.31, *p* = 0.015) was observed between centrality and ASD-related cortical difference maps (group difference, ASD-CTL, in cortical thickness). **B** Significant correlation (*r*_spin_ = 0.37, *p* = 0.003) was observed between centrality and cortical correlates of polygenic risk for ASD (association of cortical thickness and polygenic risk for ASD) maps. ASD = autism spectrum disorders, CTL = controls, PRS = polygenic risk score.

We asked whether network organization was associated with ASD-related cortical alterations (group difference in cortical thickness between ASD and CTL). For this, we used structural connectivity data (derived from diffusion-weighted tractography) from the PING dataset. Using structural connectome data, hubs (brain regions with larger degree centrality) were identified. As in earlier studies of network centrality maps in healthy individuals [26, 60], hubs were localized in the medial prefrontal, superior parietal and superior temporal regions (Fig. 1A). ASD-related cortical difference maps were obtained by computing the group difference (ASD-CTL) in cortical thickness using the ABIDE dataset (for details, see *Methods*). Consistent with previous studies [4], greater cortical thickness was observed in several brain regions including the left parietal and lateral frontal and bilateral temporal regions in individuals with ASD (Fig. 1A). Analysis of spatial similarity between ASD-related cortical difference pattern and degree centrality map was compared through correlation analysis (and statistically assessed via non-parametric spin permutation tests, see *Methods*), and revealed that ASD-related cortical difference implicated cortical hubs (*r* = 0.31, *p*_spin_ = 0.015) more strongly than non-hub regions (Fig. 1A). Cortical correlates of polygenic risk for ASD (association of cortical thickness and PRS for ASD) also implicated cortical hubs (*r* = 0.37, *p*_spin_ = 0.003) more strongly than non-hub regions (Fig. 1B).

### Hub organization is associated with cross-sectional progression of ASD-related cortical alterations as well as cortical correlates of polygenic risk for ASD

**Fig. 2:**
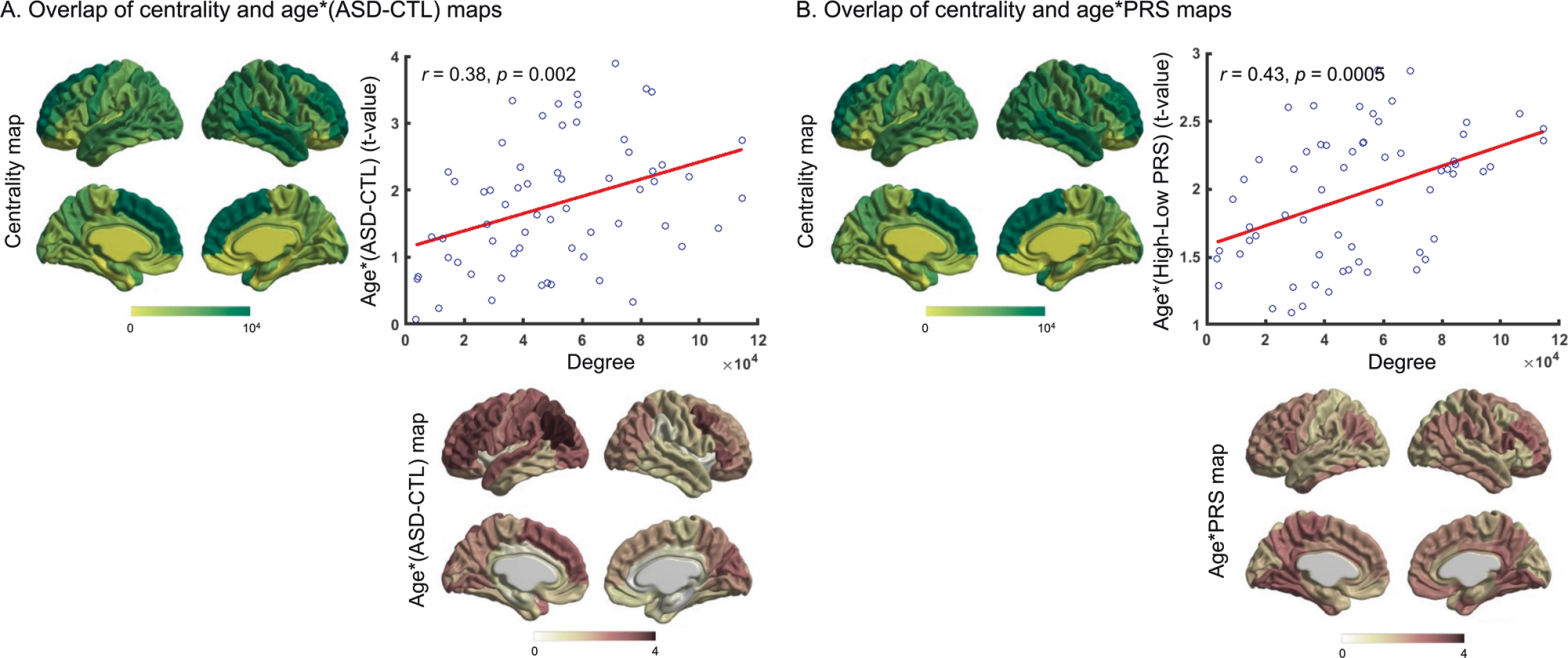
Association of structural hub organization and cross-sectional progression of ASD-related cortical alterations and cortical correlates of polygenic risk for ASD. **A** Significant correlation (*r*_spin_ = 0.38, *p* = 0.002) was observed between age interaction patterns of ASD-related cortical alterations and degree centrality maps. **B** Significant correlation (*r*_spin_ = 0.43, *p* = 0.0005) was observed between age interaction patterns of cortical correlates of polygenic risk for ASD and degree centrality maps. ASD = autism spectrum disorders, CTL = controls, PRS = polygenic risk score.

The interaction of age and difference in cortical thickness for individuals with ASD compared to controls can give insights into the cross-sectional progression of ASD-related cortical alterations. By extension, the interaction of age and cortical correlates of polygenic risk for ASD could indicate cross-sectional progression of polygenic risk-related cortical correlates. Thus, using GLMs, we first examined effect of age on (i) ASD-related cortical difference map and (ii) cortical correlates of polygenic risk for ASD. Comparison of the age interaction patterns of ASD-related cortical alterations and degree centrality maps showed significant correlations with the cortical hubs (*r* = 0.38, *p*_spin_ = 0.002, Fig. 2A). Similarly, comparison of the age interaction patterns of cortical correlates of polygenic risk for ASD and degree centrality maps showed significant correlations with the cortical hubs (*r* = 0.43, *p*_spin_ = 0.0005, Fig. 2B).

### Connectome predictors of polygenic risk for ASD relate to disease epicenters of ASD

**Fig. 3:**
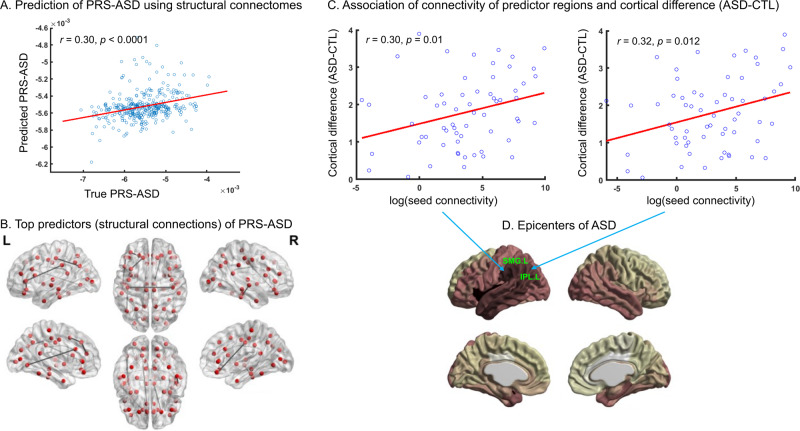
Relation of connectome predictors of PRS-ASD and disease epicenters of ASD. **A** Connectome predictive modeling using structural connectivity resulted in accurate prediction of PRS-ASD (*r* = 0.30, *p* < 0.0001 between true and predicted PRS-ASD). **B** Top predictors of the model included ipsilateral connections (the left frontal-frontal, parietal-parietal connections and right temporal-temporal connections) and bilateral postcentral connections. **C**,**D** Of these top predictors, two regions—SMG.L and IPL.L were found to be epicenters of ASD where connectivity of the region were significantly related to ASD-related cortical alterations (see *Methods*). ASD = autism spectrum disorders, CTL = controls, PRS = polygenic risk score, SMG.L = left supramarginal gyrus, IPL.L = left inferior parietal lobule, L = left hemisphere, R = right hemisphere.

Using the Connectome Predictive Modeling (CPM) framework, structural connectivity data predicted polygenic risk for ASD with *r* = 0.30, *p* < 0.0001 (Fig. 3A) suggesting that ~9% of the variance in PRS-ASD may be explained by structural connectivity. Further investigation revealed the most predictive features (brain connections) comprising of ipsilateral connections (the left frontal-frontal, parietal-parietal connections and right temporal-temporal connections) and bilateral postcentral connections (Table 2, Fig. 3B, Supplementary Fig. S1). More specifically, these connections corresponded to white matter tracts including the superior longitudinal fasciculus (connecting the inferior parietal and supramarginal gyri with the pars triangularis), the inferior longitudinal fasciculus (connecting the caudal cingulate with the lingual gyrus), the anterior commissure (connecting the temporal regions), the U-fibers (connecting the pars orbitalis and the pars triangularis, and within the frontal regions) and the callosal fibers (connecting the bilateral postcentral gyri) [45, 61–63]. We next checked whether any of these regions (with top predictive connections) were identified as epicenters of ASD. For this, as outlined in *Methods*, disease epicenters for ASD were computed (Fig. 3D). We found that two of the identified epicenters (left inferior parietal and left suparmarginal) corresponded with regions with top predictive connections (Fig. 3D). In these two regions, ASD-related cortical alterations were significantly associated with seed-based structural connectivity of the regions (Fig. 3C).

**Table 2:** Brain connections which were identified as top predictors of PRS-ASD.

Region	Region
Left Caudal Anterior Cingulate	Left Lingual
Left Pars Orbitalis	Left Pars Triangularis
Left Inferior Parietal	Left Postcentral
Left Caudal Middle Frontal	Left Superior Frontal
Left Rostral Middle Frontal	Left Superior Frontal
Left Pars Triangularis	Left Supramarginal
Left Postcentral	Right Postcentral
Right Lateral Orbito-Frontal	Right Posterior Cingulate
Right Inferior Temporal	Right Superior Temporal

*ASD* autism spectrum disorders, *PRS* polygenic risk score.

### Comparison of findings with ComBat-run cortical thickness data

We observed similar findings between our original analyses and analyses using ComBat-run cortical thickness data (details are given in Supplementary Material S4).

## Discussion

In this study, using large-scale MRI data from a clinical cohort and a general population sample, we examined whether there were links between brain network architecture and ASD-related cortical alterations. In addition, on the basis of the context of the continuum model, we also investigated whether brain network architecture also has links with cortical correlates of polygenic risk for ASD. We observed that ASD-related cortical alterations implicated cortical hubs more strongly than non-hub regions. Similarly, cortical correlates of polygenic risk for ASD also implicated cortical hubs more strongly than non-hub regions. Comparison of the age interaction patterns of ASD-related cortical alterations with degree centrality maps showed significant correlations with the cortical hubs. Similarly, comparison of the age interaction patterns of cortical correlates of polygenic risk for ASD with degree centrality maps showed significant correlations with the cortical hubs. Further investigation revealed that structural connectivity predicted polygenic risk for ASD (*r* = 0.30, *p* < 0.0001) suggesting that ~9% of the variance in polygenic risk for ASD may be explained by structural connectivity. Further investigation revealed two of the disease epicenters (the left inferior parietal and left suparmarginal) as regions with the top predictive connections. Taken together, our findings suggest a critical role of network architecture in ASD-related cortical alterations and in cortical correlates related to polygenic risk for ASD.

Several studies have consistently shown cortical alterations in individuals with ASD [1–10]. Our findings add to the extant literature of ASD research by revealing how brain network architecture is linked with the cortical alterations associated with ASD. Firstly, we observed that regions with more hub characteristics (greater centrality) showed greater ASD-related cortical alterations, in consistent with previous studies that have implicated hubs in brain disorders [23, 27–29]. Secondly, in consistent with previous studies [23, 33], we observed that brain regions with the largest ASD-related cortical alterations denote disease epicenters for ASD (Fig. 3D). Thirdly, we observed that brain regions with high degree centrality exhibited greater cross-sectional progression of ASD-related cortical alterations (Fig. 2D).

The epicenters identified in our study encompass somatosensory, motor and visual areas along with regions involved in auditory processing and low-level sensory integration (Fig. 3D). Individuals with ASD usually show impairments in motor behaviors, responses to tactile, auditory, and visual stimuli, and in their processing of language and nonlinguistic social stimuli [64–68]. More interestingly, the two epicenters (suparmarginal gyrus and inferior parietal lobule) identified as top predictors of polygenic risk for ASD are involved in visuospatial processing. These brain regions, located at the intersection of the visual, auditory and somatosensory cortices, comprise of neurons with multimodal properties which can process several stimuli concurrently. Inferior parietal lobule is involved in integration and interpretation of sensory information, emotional perception of sensory stimuli. The inferior parietal lobule, with its connections to both Broca’s area and Wernicke’s area, may serve as information relay center between these areas for language-related functions. The inferior parietal lobule, part of the social brain system, is also involved in social perception [69] and executive attention [70]. Not surprisingly, alterations in the connectivity of the inferior parietal lobule are linked with deficits in social cognition in ASD [1, 71]. Lastly, altered white matter in the inferior parietal lobule has been shown in children with ASD [72] which in turn has been associated with impaired motor performance [73].

The mechanisms behind the influence of brain network architecture on ASD-related cortical alterations are not clear. In terms of cortical alterations, several factors including larger neurons, increased number of neurons [74], greater microglial cell density and somal volume [75] and greater number of synaptic spines and reduced developmental synaptic pruning [76] have been associated with increased cortical thickness in individuals with ASD. On the other hand, alterations in white matter connectivity in ASD might be due to increased packing density [77], increased oedema from inflammation [78] and reduced thickness of myelin [79]. In addition, reduced synaptic pruning seen in children with ASD might hinder axonal remodeling resulting in altered connectivity [76, 80]. Further, abnormalities in social perception and executive attention (which are considered broad phenotypes for ASD) might impact processing of neural information, which in turn might alter brain structure and connectivity [81].

Our findings that network architecture is linked to ASD-related cortical alterations in clinical cohort as well as cortical correlates of ASD polygenic risk in general population highlight an emerging consensus that psychiatric disorders including ASD may be viewed as continuum models as opposed to conventional diagnostic groups. Traditionally, psychiatric disorders are categorized as diagnostic groups: *individuals with disorder (case)* and *healthy individuals (control)*. However, recent studies have indicated that psychiatric disorders may be viewed as a continuum with a normal distribution of psychiatric tendencies in the general population, where a full diagnosis is at the severe tail of the distribution [12, 13, 15–21]. Evidence toward this has come from behavioral and imaging studies. For instance, autistic traits (e.g., social and communications deficits) extend beyond diagnostic groups into the general population [19]. In terms of imaging, brain alterations have been observed not only for case-control differences [6–9, 82], but also for autistic traits in the general population [11–14]. One such study found significant negative association of cortical thickness (in the right superior temporal cortex) and a continuous measure of autistic traits in a large longitudinal sample of normally developing youths [83]. In another study, Blanken et al. observed significant association of gyrification and autistic traits along a continuum in a large population-based sample of children [12]. Recently, we found that cortical correlates of polygenic risk for ASD in general population overlap with the cortical alterations seen in individuals with ASD [22]. Extending these previous studies, our findings indicate that brain network architecture is linked to ASD-related cortical alterations in clinical population as well as cortical correlates of ASD polygenic risk in general population. Our observation that structural connectivity could accurately predict polygenic risk for ASD and more importantly, that two of the top predictor regions were identified as disease epicenters of ASD lends more credence on the continuum model of ASD spanning from healthy individuals with genetic risk to individuals with ASD.

In conclusion, our study highlights a critical role of network architecture in ASD-related cortical alterations and in cortical correlates of polygenic risk for ASD. Our study underscores the need for investigating PRS in addition to multi-modal neuroimaging measures to better understand the interplay between genetic risk and brain abnormalities associated with ASD.

## Supplementary information

Supplementary Material

## Data Availability

The ABIDE dataset is freely available to researchers via NITRC (https://fcon_1000.projects.nitrc.org/indi/abide/). The PING dataset is available to researchers with data user agreement via NDAR (https://nda.nih.gov/edit_collection.html?id=2607).

## Code availability

Codes used in the study were performed using SurfStat (https://www.math.mcgill.ca/keith/surfstat/) in addition to other toolboxes in MATLAB (version 2019b). The codes are available from the authors upon request.

## References

[1] Hadjikhani N, Joseph RM, Snyder J, Tager-Flusberg H (2006). Anatomical differences in the mirror neuron system and social cognition network in autism. Cereb Cortex.

[2] Hyde KL, Samson F, Evans AC, Mottron L (2010). Neuroanatomical differences in brain areas implicated in perceptual and other core features of autism revealed by cortical thickness analysis and voxel-based morphometry. Hum Brain Mapp.

[3] Wallace GL, Dankner N, Kenworthy L, Giedd JN, Martin A (2010). Age-related temporal and parietal cortical thinning in autism spectrum disorders. Brain.

[4] Khundrakpam BS, Lewis JD, Kostopoulos P, Carbonell F, Evans AC (2017). Cortical Thickness Abnormalities in Autism Spectrum Disorders Through Late Childhood, Adolescence, and Adulthood: A Large - Scale MRI Study. Cereb Cortex.

[5] Zielinski BA, Prigge MBD, Nielsen JA, Froehlich AL, Abildskov TJ, Anderson JS (2014). Longitudinal changes in cortical thickness in autism and typical development. Brain.

[6] Lange N, Travers BG, Bigler ED, Prigge MBD, Froehlich AL, Nielsen JA (2015). Longitudinal volumetric brain changes in autism spectrum disorder ages 6-35 years. Autism Res.

[7] Van Rooij D, Anagnostou E, Arango C, Auzias G, Behrmann M, Busatto GF (2018). Cortical and subcortical brain morphometry differences between patients with autism spectrum disorder and healthy individuals across the lifespan: Results from the ENIGMA ASD working group. Am J Psychiatry.

[8] Raznahan A, Toro R, Daly E, Robertson D, Murphy C, Deeley Q (2010). Cortical anatomy in autism spectrum disorder: an in vivo MRI study on the effect of age. Cereb Cortex.

[9] Ecker C, Ronan L, Feng Y, Daly E, Murphy C, Ginestet CE (2013). Intrinsic gray-matter connectivity of the brain in adults with autism spectrum disorder. Proc Natl Acad Sci.

[10] Bezgin G, Lewis JD, Evans AC (2018). Developmental changes of cortical white-gray contrast as predictors of autism diagnosis and severity. Transl Psychiatry.

[11] Barnea-Goraly N, Lotspeich LJ, Reiss AL (2010). Similar White Matter Aberrations in Children With Autism and Their Unaffected Siblings. Arch Gen Psychiatry.

[12] Blanken LME, Mous SE, Ghassabian A, Muetzel RL, Schoemaker NK, El Marroun H (2015). Cortical morphology in 6- to 10-year old children with autistic traits: A population-based neuroimaging study. Am J Psychiatry.

[13] Blanken LME, Muetzel RL, Jaddoe VWV, Verhulst FC, van der Lugt A, Tiemeier H (2017). White matter microstructure in children with autistic traits. Psychiatry Res - Neuroimaging.

[14] Di Martino A, Shehzad Z, Kelly C, Roy AK, Gee DG, Uddin LQ (2009). Relationship between cingulo-insular functional connectivity and autistic traits in neurotypical adults. Am J Psychiatry.

[15] Wakabayashi A, Baron-Cohen S, Wheelwright S (2006). Are autistic traits an independent personality dimension? A study of the Autism-Spectrum Quotient (AQ) and the NEO-PI-R. Pers Individ Dif.

[16] Robinson EB, St Pourcain B, Anttila V, Kosmicki JA, Bulik-Sullivan B, Grove J (2016). Genetic risk for autism spectrum disorders and neuropsychiatric variation in the general population. Nat Genet.

[17] Plomin R, Haworth CMA, Davis OSP (2009). Common disorders are quantitative traits. Nat Rev Genet.

[18] Robinson EB, Koenen KC, McCormick MC, Munir K, Hallett V, Happé F (2011). Evidence That Autistic Traits Show the Same Etiology in the General Population and at the Quantitative Extremes (5%, 2.5%, and 1%). Arch Gen Psychiatry..

[19] Constantino JN, Todd RD (2003). Autistic Traits in the General Population. Arch Gen Psychiatry.

[20] Constantino JN (2011). The quantitative nature of autistic social impairment. Pediatr Res.

[21] Hyseni F, Blanken LME, Muetzel R, Verhulst FC, Tiemeier H, White T (2019). Autistic traits and neuropsychological performance in 6- to-10-year-old children: a population-based study. Child Neuropsychol.

[22] Khundrakpam B, Vainik U, Gong J, Al-Sharif N, Bhutani N, Kiar G, et al. Neural correlates of polygenic risk score for autism spectrum disorders in general population. Brain Commun. 2020. 10.1093/braincomms/fcaa092. 10.1093/braincomms/fcaa092 PMC7475696 32954337

[23] Larivière S, Rodríguez-Cruces R, Royer J, Caligiuri ME, Gambardella A, Concha L, et al. Network-based atrophy modeling in the common epilepsies: A worldwide ENIGMA study. Sci Adv. 2020;6:eabc6457. 10.1126/sciadv.abc6457 PMC7673818 33208365

[24] Zhou J, Gennatas ED, Kramer JH, Miller BL, Seeley WW (2012). Predicting Regional Neurodegeneration from the Healthy Brain Functional Connectome. Neuron.

[25] Bullmore E, Sporns O (2009). Complex brain networks: Graph theoretical analysis of structural and functional systems. Nat Rev Neurosci.

[26] van den Heuvel MP, Sporns O (2013). Network hubs in the human brain. Trends Cogn Sci.

[27] Crossley NA, Mechelli A, Scott J, Carletti F, Fox PT, Mcguire P (2014). The hubs of the human connectome are generally implicated in the anatomy of brain disorders. Brain.

[28] Fornito A, Zalesky A, Breakspear M (2015). The connectomics of brain disorders. Nat Rev Neurosci.

[29] Baker STE, Lubman DI, Yucel M, Allen NB, Whittle S, Fulcher BD (2015). Developmental Changes in Brain Network Hub Connectivity in Late Adolescence. J Neurosci.

[30] Buckner RL, Sepulcre J, Talukdar T, Krienen FM, Liu H, Hedden T (2009). Cortical Hubs Revealed by Intrinsic Functional Connectivity: Mapping, Assessment of Stability, and Relation to Alzheimer’s Disease. J Neurosci.

[31] Filippi M, Basaia S, Canu E, Imperiale F, Magnani G, Falautano M (2020). Changes in functional and structural brain connectome along the Alzheimer’s disease continuum. Mol Psychiatry.

[32] Shafiei G, Markello RD, Makowski C, Talpalaru A, Kirschner M, Devenyi GA (2020). Spatial Patterning of Tissue Volume Loss in Schizophrenia Reflects Brain Network Architecture. Biol Psychiatry.

[33] Zeighami Y, Ulla M, Iturria-Medina Y, Dadar M, Zhang Y, Larcher KMH (2015). Network structure of brain atrophy in de novo parkinson’s disease. Elife.

[34] Di Martino A, Yan CG, Li Q, Denio E, Castellanos FX, Alaerts K (2014). The autism brain imaging data exchange: towards a large-scale evaluation of the intrinsic brain architecture in autism. Mol Psychiatry.

[35] Jernigan TL, Brown TT, Hagler DJ, Akshoomoff N, Bartsch H, Newman E (2016). The Pediatric Imaging, Neurocognition, and Genetics (PING) Data Repository. Neuroimage.

[36] Sherif T, Rioux P, Rousseau M-E, Kassis N, Beck N, Adalat R (2014). CBRAIN: a web-based, distributed computing platform for collaborative neuroimaging research. Front Neuroinform.

[37] McCarthy S, Das S, Kretzschmar W, Delaneau O, Wood AR, Teumer A (2016). A reference panel of 64,976 haplotypes for genotype imputation. Nat Genet.

[38] Chang CC, Chow CC, Tellier LC, Vattikuti S, Purcell SM, Lee JJ (2015). Second-generation PLINK: rising to the challenge of larger and richer datasets. Gigascience.

[39] Euesden J, Lewis CM, O’Reilly PF (2015). PRSice: Polygenic Risk Score software. Bioinformatics.

[40] Grove J, Ripke S, Als TD, Mattheisen M, Walters RK, Won H (2019). Identification of common genetic risk variants for autism spectrum disorder. Nat Genet.

[41] Jenkinson M, Beckmann CF, Behrens TEJ, Woolrich MW, Smith SM. FSL. Neuroimage. 2012;62:782–90. 10.1016/j.neuroimage.2011.09.015 21979382

[42] Behrens TEJ, Woolrich MW, Jenkinson M, Johansen-Berg H, Nunes RG, Clare S (2003). Characterization and Propagation of Uncertainty in Diffusion-Weighted MR Imaging. Magn Reson Med.

[43] Behrens TEJ, Berg HJ, Jbabdi S, Rushworth MFS, Woolrich MW (2007). Probabilistic diffusion tractography with multiple fibre orientations: What can we gain?. Neuroimage.

[44] Sporns O, Honey CJ, Kötter R (2007). Identification and classification of hubs in brain networks. PLoS ONE.

[45] Lin YH, Dhanaraj V, Mackenzie AE, Young IM, Tanglay O, Briggs RG (2021). Anatomy and White Matter Connections of the Parahippocampal Gyrus. World Neurosurg.

[46] Larivière S, Rodríguez-Cruces R, Royer J, Caligiuri ME, Gambardella A, Concha L (2020). Network-based atrophy modeling in the common epilepsies: a worldwide ENIGMA study. Sci Adv.

[47] Khundrakpam BS, Reid A, Brauer J, Carbonell F, Lewis J, Ameis S (2013). Developmental changes in organization of structural brain networks. Cereb Cortex.

[48] Moradi E, Khundrakpam B, Lewis JD, Evans AC, Tohka J (2017). Predicting symptom severity in autism spectrum disorder based on cortical thickness measures in agglomerative data. Neuroimage.

[49] Valk SL, Di Martino A, Milham MP, Bernhardt BC (2015). Multicenter mapping of structural network alterations in autism. Hum Brain Mapp.

[50] Hamer D, Sirota L (2000). Beware the chopsticks gene. Mol Psychiatry.

[51] Fortin J-P, Cullen N, Sheline YI, Taylor WD, Aselcioglu I, Cook PA (2018). Harmonization of cortical thickness measurements across scanners and sites. Neuroimage.

[52] Alexander-Bloch AF, Shou H, Liu S, Satterthwaite TD, Glahn DC, Shinohara RT, et al. On testing for spatial correspondence between maps of human brain structure and function. Neuroimage. 2018. 2018. 10.1016/j.neuroimage.2018.05.070. 10.1016/j.neuroimage.2018.05.070 PMC6095687 29860082

[53] Reardon PK, Seidlitz J, Vandekar S, Liu S, Patel R, Park MTM (2018). Normative brain size variation and brain shape diversity in humans. Science.

[54] Finn ES, Shen X, Scheinost D, Rosenberg MD, Huang J, Chun MM (2015). Functional connectome fingerprinting: Identifying individuals using patterns of brain connectivity. Nat Neurosci.

[55] Shen X, Finn ES, Scheinost D, Rosenberg MD, Chun MM, Papademetris X (2017). Using connectome-based predictive modeling to predict individual behavior from brain connectivity. Nat Protoc.

[56] Wang Z, Goerlich KS, Ai H, Aleman A, Luo YJ, Xu P. Connectome-based predictive modeling of individual anxiety. Cereb Cortex. 2021;31:3006–20. 10.1093/cercor/bhaa407 33511990

[57] Yoo K, Rosenberg MD, Hsu WT, Zhang S, Li CSR, Scheinost D (2018). Connectome-based predictive modeling of attention: Comparing different functional connectivity features and prediction methods across datasets. Neuroimage.

[58] Rutherford HJV, Potenza MN, Mayes LC, Scheinost D (2020). The Application of Connectome-Based Predictive Modeling to the Maternal Brain: Implications for Mother-Infant Bonding. Cereb Cortex.

[59] Xia M, Wang J, He Y (2013). BrainNet Viewer: A Network Visualization Tool for Human Brain Connectomics. PLoS ONE.

[60] Zuo XN, Ehmke R, Mennes M, Imperati D, Castellanos FX, Sporns O (2012). Network centrality in the human functional connectome. Cereb Cortex.

[61] Barbeau EB, Descoteaux M, Petrides M (2020). Dissociating the white matter tracts connecting the temporo-parietal cortical region with frontal cortex using diffusion tractography. Sci Rep..

[62] Burks JD, Boettcher LB, Conner AK, Glenn CA, Bonney PA, Baker CM (2017). White matter connections of the inferior parietal lobule: A study of surgical anatomy. Brain Behav.

[63] Schmahmann JD, Smith EE, Eichler FS, Filley CM (2008). Cerebral White Matter. Ann N. Y Acad Sci.

[64] Schultz RT (2005). Developmental deficits in social perception in autism: The role of the amygdala and fusiform face area. Int J Dev Neurosci.

[65] Schultz RT, Grelotti DJ, Klin A, Kleinman J, Van Der Gaag C, Marois R (2003). The role of the fusiform face area in social cognition: Implications for the pathobiology of autism. Philos Trans R Soc B Biol Sci.

[66] Tager-Flusberg H (1981). On the nature of linguistic functioning in early infantile autism. J Autism Dev Disord.

[67] Redcay E (2008). The superior temporal sulcus performs a common function for social and speech perception: Implications for the emergence of autism. Neurosci Biobehav Rev.

[68] Leekam SR, Nieto C, Libby SJ, Wing L, Gould J (2007). Describing the sensory abnormalities of children and adults with autism. J Autism Dev Disord.

[69] Pelphrey KA, Carter EJ. Brain mechanisms for social perception: lessons from autism and typical development. Ann N Y Acad Sci. Blackwell Publishing Inc. 2008;1145:283–99. 10.1196/annals.1416.007 PMC2804066 19076404

[70] Corbetta M, Patel G, Shulman GL (2008). The reorienting system of the human brain: from environment to theory of mind. Neuron.

[71] Cheng Y, Chou KH, Fan YT, Lin CP. ANS: Aberrant neurodevelopment of the social cognition network in adolescents with autism spectrum disorders. PLoS ONE. 2011;6:e18905. 10.1371/journal.pone.0018905. 10.1371/journal.pone.0018905 PMC3082537 21541322

[72] Yang Q, Huang P, Li C, Fang P, Zhao N, Nan J (2018). Mapping alterations of gray matter volume and white matter integrity in children with autism spectrum disorder: Evidence from fMRI findings. Neuroreport.

[73] Hanaie R, Mohri I, Kagitani-Shimono K, Tachibana M, Matsuzaki J, Hirata I (2016). White matter volume in the brainstem and inferior parietal lobule is related to motor performance in children with autism spectrum disorder: A voxel-based morphometry study. Autism Res.

[74] Courchesne E, Mouton PR, Calhoun ME, Semendeferi K, Ahrens-Barbeau C, Hallet MJ (2011). Neuron number and size in prefrontal cortex of children with autism. JAMA.

[75] Morgan JT, Chana G, Pardo CA, Achim C, Semendeferi K, Buckwalter J (2010). Microglial activation and increased microglial density observed in the dorsolateral prefrontal cortex in autism. Biol Psychiatry.

[76] Tang G, Gudsnuk K, Kuo SH, Cotrina ML, Rosoklija G, Sosunov A (2014). Loss of mTOR-dependent macroautophagy causes autistic-like synaptic pruning deficits. Neuron.

[77] Bauman ML, Kemper TL (2005). Neuroanatomic observations of the brain in autism: a review and future directions. Int J Dev Neurosci.

[78] Vargas DL, Nascimbene C, Krishnan C, Zimmerman AW, Pardo CA (2005). Neuroglial activation and neuroinflammation in the brain of patients with autism. Ann Neurol.

[79] Zikopoulos B, Barbas H (2010). Changes in Prefrontal Axons May Disrupt the Network in Autism. J Neurosci.

[80] Auerbach BD, Osterweil EK, Bear MF (2011). Mutations causing syndromic autism define an axis of synaptic pathophysiology. Nature.

[81] Valla JM, Belmonte MK (2013). Detail-oriented cognitive style and social communicative deficits, within and beyond the autism spectrum: Independent traits that grow into developmental interdependence. Dev Rev.

[82] Khundrakpam BS, Lewis JD, Reid A, Karama S, Zhao L, Chouinard-Decorte F (2017). Imaging structural covariance in the development of intelligence. Neuroimage.

[83] Wallace GL, Shaw P, Lee NR, Clasen LS, Raznahan A, Lenroot RK (2012). Distinct cortical correlates of autistic versus antisocial traits in a longitudinal sample of typically developing youth. J Neurosci.

