# Supplementary material for: A critical role of brain network architecture in a continuum model of autism spectrum disorders spanning from healthy individuals with genetic liability to individuals with ASD
Source: Mol Psychiatry. 2022 Dec 27;28(3):1210–8. doi: 10.1038/s41380-022-01916-w (PMC10005951; doi:10.1038/s41380-022-01916-w)

**Supplementary Material**

***S1 – Image acquisition:***

For PING: A standardized multi-modal high-resolution structural MRI protocol comprising of 3D T1-weighted volumes and a set of diffusion-weighted scans was used across 9 sites and 12 3T scanners (Jernigan et al. 2016). Across the nine sites and 12 scanners, a standardized multiple-modality high-resolution structural MRI protocol was applied involving 3D T1- and T2-weighted volumes and a set of diffusion-weighted scans. The protocol included a conventional three-plane localizer, a sagittal 3D inversion recovery spoiled gradient echo T1-weighted volume optimized for maximum gray/white matter contrast (echo time = 3.5 ms, repetition time = 8.1 ms, inversion time = 640 ms, flip angle = 8°, receiver bandwidth = ±31.25 kHz, FOV = 24 cm, frequency = 256, phase = 192, slice thickness = 1.2 mm), and two axial 2D diffusion tensor imaging (DTI) PEpolar scans (30-directions bvalue = 1,000, TE = 83 ms, TR = 13,600 ms, frequency = 96, phase = 96, slice thickness = 2.5 mm).

For ABIDE: The ABIDE database is an agglomerated dataset of 1112 resting-state functional magnetic resonance imaging (rsfMRI) data sets openly shared from 17 sites with corresponding structural MRI and phenotypic information from 539 individuals with ASDs and 573 age-matched typical controls (<http://fcon_1000.projects.nitrc.org/indi/abide/>) (Di Martino et al. 2014). The imaging protocols differ across the 17 sites, and as such, have not been included in the manuscript.

***S2 – Quality Control:***

We made particular efforts to minimize the confound of the multiple ABIDE sites by performing a stringent quality control (QC) procedure using both automated and visual inspections. Automated QC was based on outputs from CIVET (summary statistics of e.g. number of surface-surface intersections) which allowed identification of outliers. In parallel, visual inspection was performed by two independent reviewers and only scans with consensus of the two reviewers were used. Exclusion criteria for QC procedure include - data with motion artifacts, a low signal to noise ratio, artifacts due to hyperintensities from blood vessels, surface-surface intersections, or poor placement of the grey or white matter (GM and WM) surface for any reason.


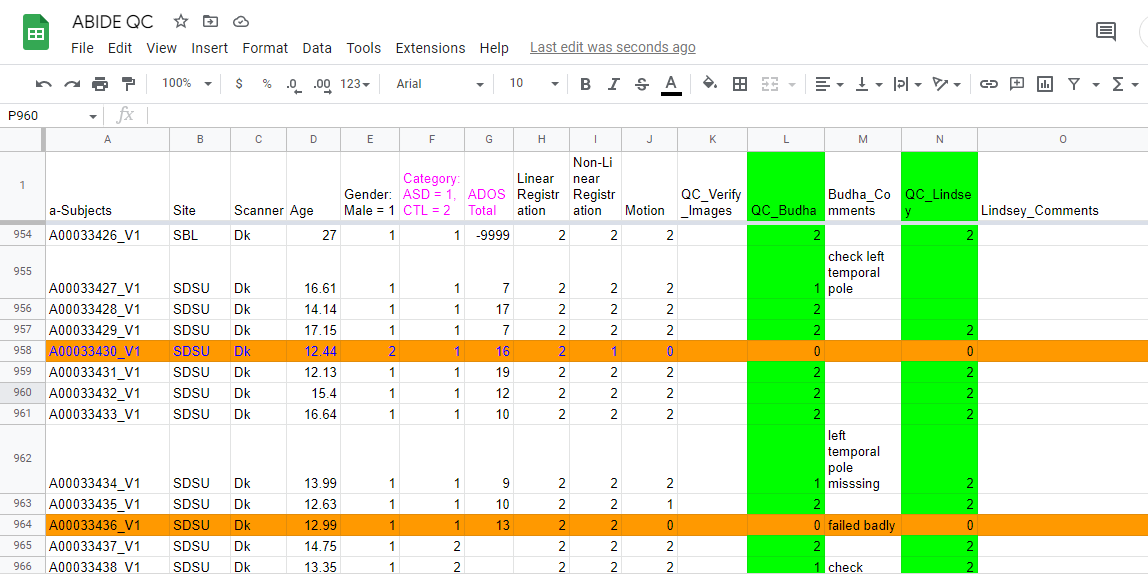


ABIDE being an agglomerated dataset, contained confounds. One of the major confounds we encountered was the presence of blood vessels in several scans of a particular site with severe consequences (e.g. large number of surface-surface intersections) as shown below –
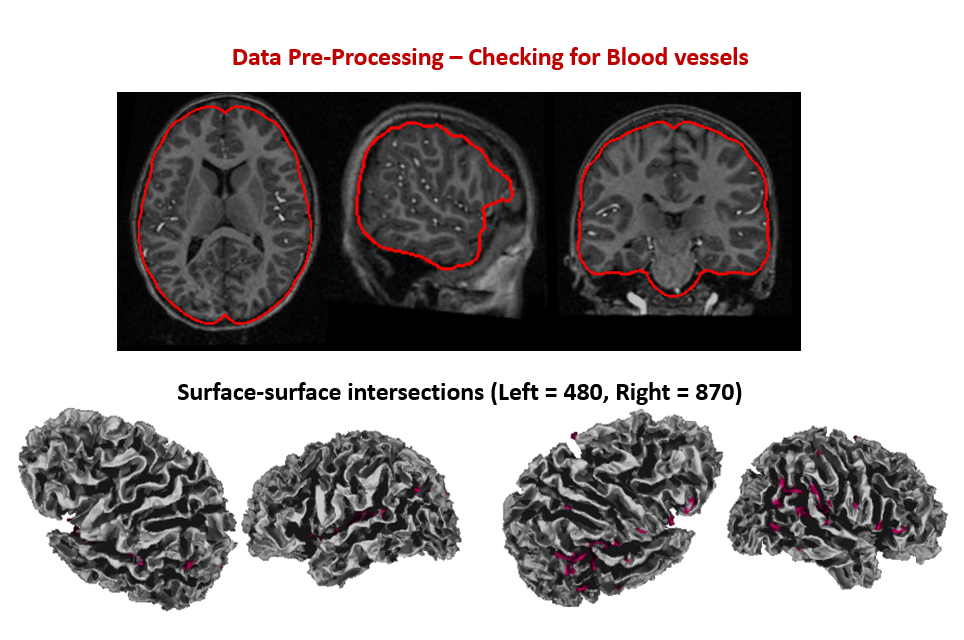


These scans with blood vessels were subsequently marked as QC ‘failed’ and were not used in the analysis.

***S3 – Markov Chain Monte Carlo sampling:***

Traditional sampling techniques are very slow especially in high-dimensional parameter spaces because samples are proposed at random due to which each has a very small chance of acceptance. Markov Chain Monte Carlo (MCMC) is a sampling technique that that addresses this problem by proposing samples preferentially in areas of high probability (Behrens et al. 2003, 2007). The high probability of accepting samples allows for many samples to be drawn and allows computation of the posterior probability density function (pdf) in a relatively short period of time. As in (Behrens et al. 2003), model estimation was performed using MCMC after analytically marginalizing over variance parameters. Markov chains were burned in for 2000 jumps, and then ran for a further 1000 jumps sampling every 20. Initialization is using the log-linear diffusion tensor fit. If a preprocessed neighbouring voxel contains more than one surviving fiber orientation, the parameters are initialized based on the mean values from this neighbouring voxel. This procedure resulted in samples from the posterior probability distribution on every parameter in the model, including the orientation and volume fraction parameters from each fiber population.

***S4 – Comparison of findings with ComBat-run cortical thickness data:***

We observed a strong, significant positive correlation (*r =* 0.99, *p* < 0.0001) between the original cortical thickness (CT_ori) and combat-run cortical thickness (CT_combat) values for the PING dataset. For the ABIDE dataset, we also observed a strong, significant positive correlation (*r =* 0.97, *p* < 0.0001) between the original cortical thickness (CT_ori) and combat-run cortical thickness (CT_combat) values. We observed similar results for the analyses e.g. similar patterns of effect of (ASD-CTL) on cortical thickness using CT_ori and CT_combat for ABIDE dataset –

**(ASD-CTL) on CT_ori (ASD-CTL) on CT_combat**


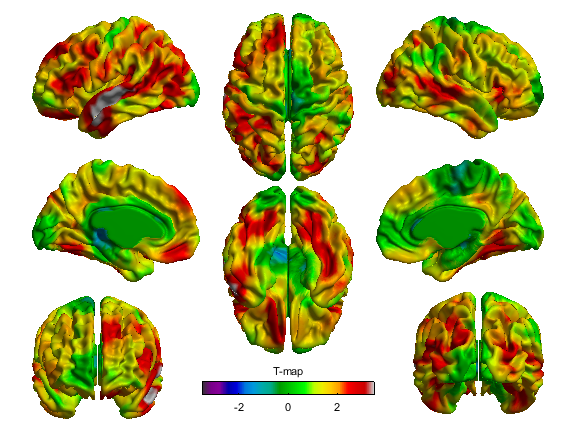

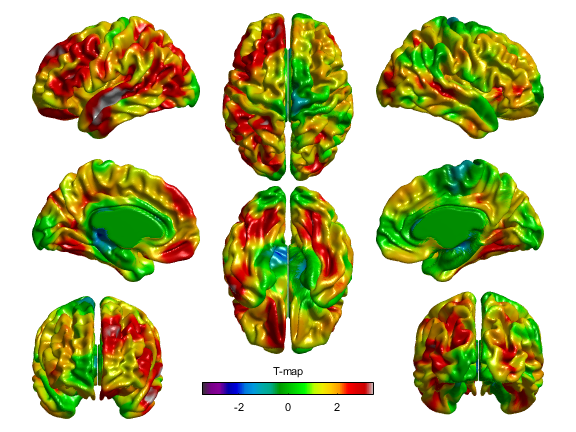


Similarly, for PING dataset, we observed similar patterns of effect of PRS on cortical thickness using CT_ori and CT_combat –

**PRS and CT_ori PRS and CT_combat**


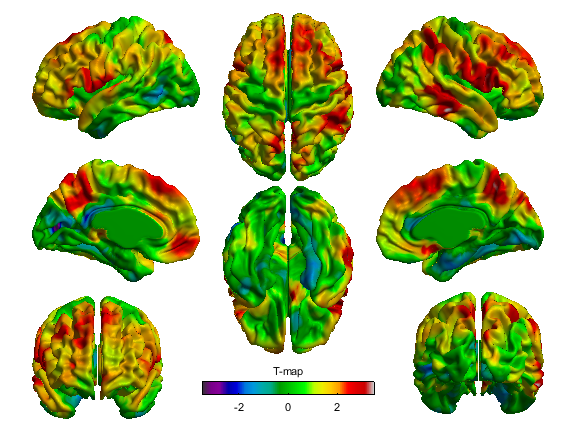

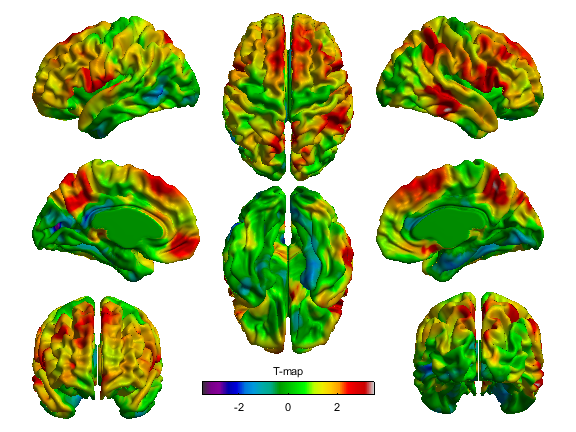


Not surprisingly, we observed similar results as **Figure 1** in our Manuscript i.e. statistically significant overlap (using spin permutation test) of *i*) degree centrality and (ASD-CTL) in cortical thickness (using CT_combat) for ABIDE, and *ii*) degree centrality and effect of PRS on cortical thickness (using CT_combat) for PING dataset. As compared to our original findings of *r*=0.31, *p*=0.015 for significant overlap of degree centrality and (ASD-CTL) in cortical thickness (using CT_ori, **Figure 1A** in Manuscript), we observed *r*=0.32, *p*=0.011 for significant overlap of degree centrality and (ASD-CTL) in cortical thickness (using CT_combat). As compared to our original findings of *r*=0.37, *p*=0.003 for significant overlap of degree centrality and effect of PRS on cortical thickness (using CT_ori, **Figure 1B** in Manuscript), we observed *r*=0.38, *p*=0.002 for significant overlap of degree centrality and effect of PRS on cortical thickness (using CT_combat).

**Overlap of centrality and (ASD-CTL) in CT_combat Overlap of centrality and PRS & CT_combat**


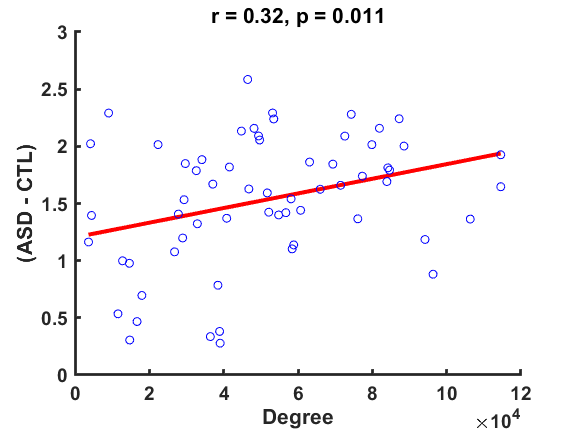

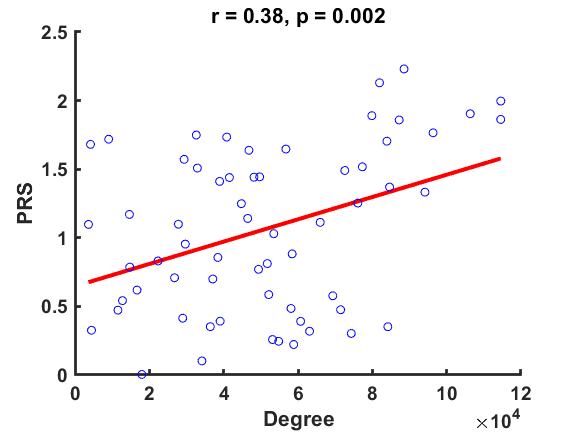


Thus, we observed high similarities between our original analyses and analyses using combat-run cortical thickness values.

***S5 – Regions of Interest (ROI):***

| **Left hemisphere** | **Right hemisphere** |
| --- | --- |
| lh.caudalanteriorcingulate | rh.caudalanteriorcingulate |
| lh.caudalmiddlefrontal | rh.caudalmiddlefrontal |
| lh.cuneus | rh.cuneus |
| lh.entorhinal | rh.entorhinal |
| lh.fusiform | rh.fusiform |
| lh.inferiorparietal | rh.inferiorparietal |
| lh.inferiortemporal | rh.inferiortemporal |
| lh.insula | rh.insula |
| lh.isthmuscingulate | rh.isthmuscingulate |
| lh.lateraloccipital | rh.lateraloccipital |
| lh.lateralorbitofrontal | rh.lateralorbitofrontal |
| lh.lingual | rh.lingual |
| lh.medialorbitofrontal | rh.medialorbitofrontal |
| lh.middletemporal | rh.middletemporal |
| lh.paracentral | rh.paracentral |
| lh.parahippocampal | rh.parahippocampal |
| lh.parsopercularis | rh.parsopercularis |
| lh.parsorbitalis | rh.parsorbitalis |
| lh.parstriangularis | rh.parstriangularis |
| lh.pericalcarine | rh.pericalcarine |
| lh.postcentral | rh.postcentral |
| lh.posteriorcingulate | rh.posteriorcingulate |
| lh.precentral | rh.precentral |
| lh.precuneus | rh.precuneus |
| lh.rostralanteriorcingulate | rh.rostralanteriorcingulate |
| lh.rostralmiddlefrontal | rh.rostralmiddlefrontal |
| lh.superiorfrontal | rh.superiorfrontal |
| lh.superiorparietal | rh.superiorparietal |
| lh.superiortemporal | rh.superiortemporal |
| lh.supramarginal | rh.supramarginal |
| lh.transversetemporal | rh.transversetemporal |

***S6 – Histogram plots of data:***

**ABIDE data**

***
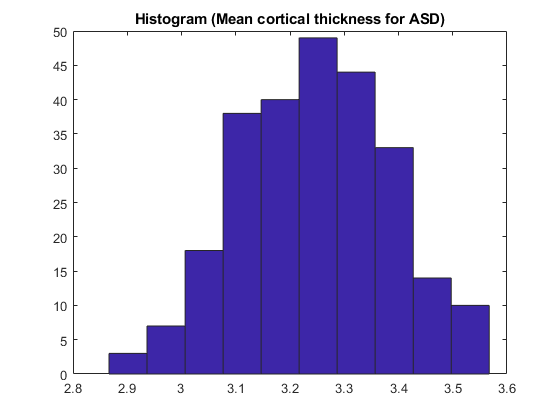

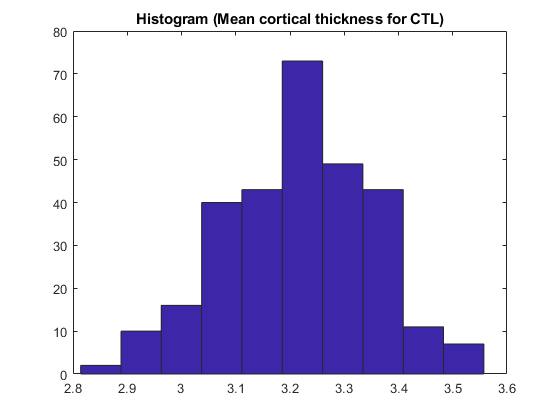
***

**PING data**

***
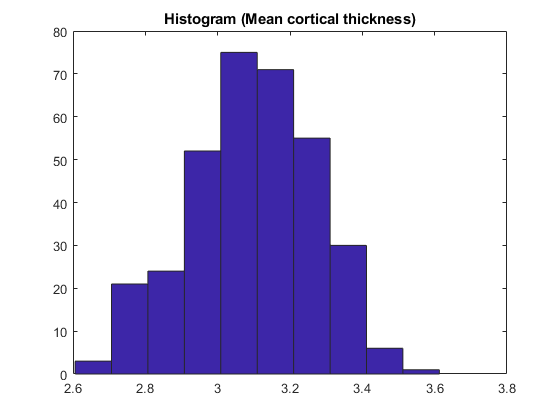

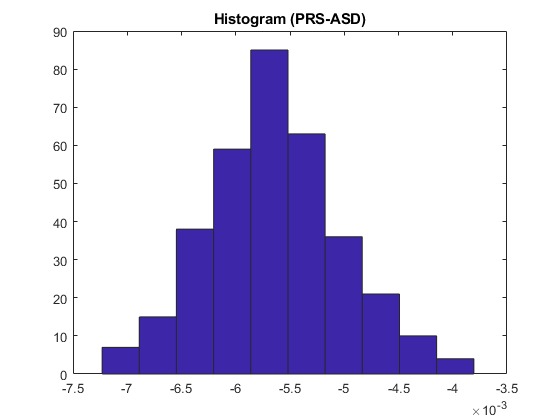
***

***Figure S1 – Top predictors (structural connections) of PRS for ASD:***


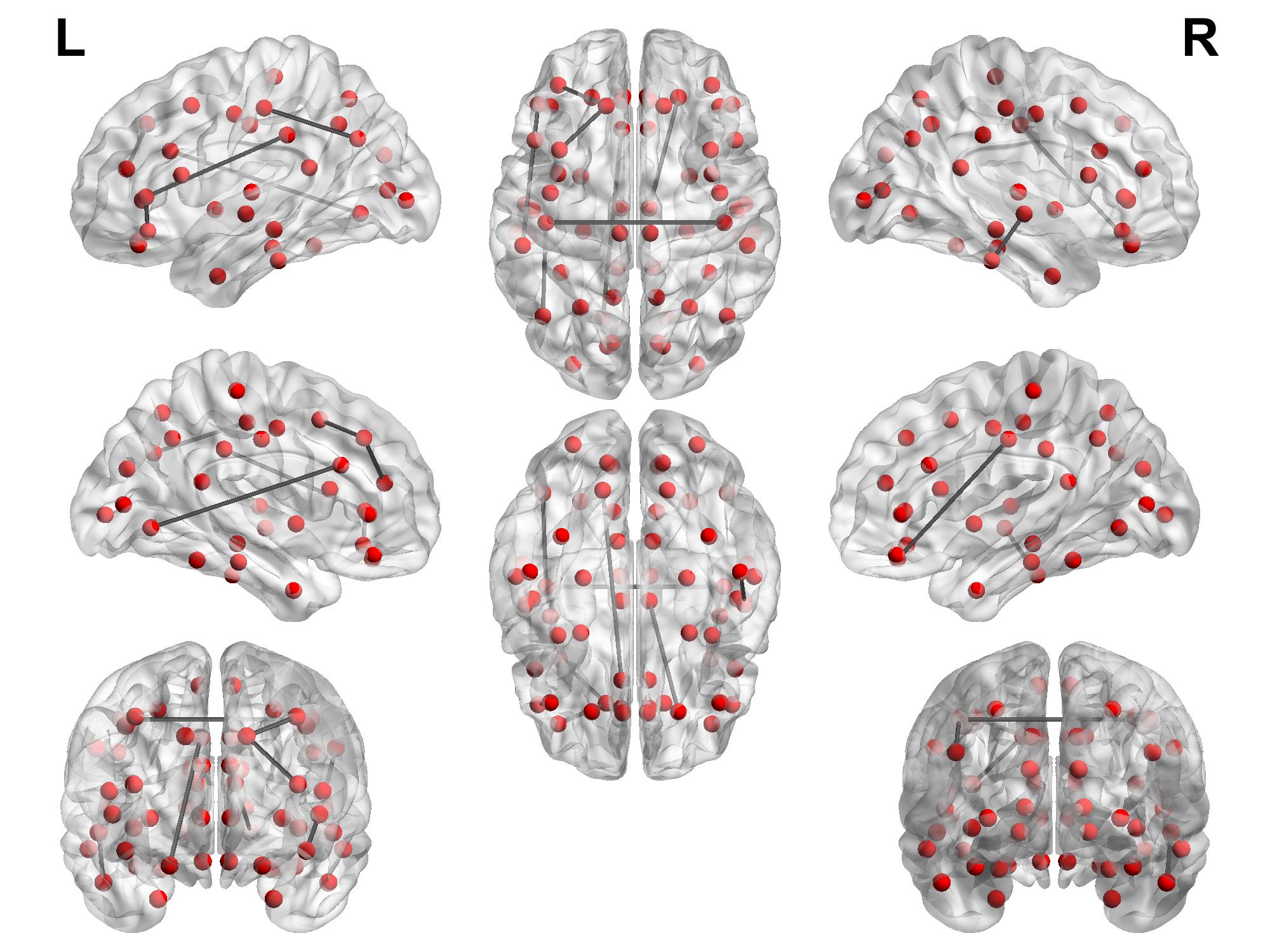

Supplement: Supplementary file 1 — Supplementary Material [file 41380_2022_1916_MOESM1_ESM.docx]
